# Prospective evaluation of biodegradable polymeric sealant for intraoperative air leaks

**DOI:** 10.1186/s13019-016-0563-3

**Published:** 2016-12-12

**Authors:** Bernard J. Park, John M. Snider, Nathan R. Bates, Stephen D. Cassivi, G. Kimble Jett, Joshua R. Sonett, Eric M. Toloza

**Affiliations:** 1 Memorial Sloan Kettering Cancer Center, 1275 York Avenue, Box 531, New York, NY 10065 USA; 2 Mercy Health System, Janesville, WI USA; 3 Cardiothoracic & Vascular Surgical Associates, Jacksonville, FL USA; 4 Mayo Clinic, Rochester, MN USA; 5 The Heart Hospital Baylor Plano, Plano, TX USA; 6 New York-Presbyterian Hospital, Columbia University, New York, NY USA; 7 H. Lee Moffitt Cancer Center and Research Institute, Tampa, FL USA

**Keywords:** Lung cancer, Lung surgery, Video-assisted thoracic surgery, Robotic surgery, Intraoperative air leak, Postoperative air leak, Pleural air leak sealant

## Abstract

**Background:**

A biodegradable polymeric sealant has been previously shown to reduce postoperative air leaks after open pulmonary resection. The aim of this study was to evaluate safety and efficacy during minimally invasive pulmonary resection.

**Methods:**

In a multicenter prospective single-arm trial, 112 patients with a median age of 69 years (range 34–87 years) were treated with sealant for at least one intraoperative air leak after standard methods of repair (sutures, staples or cautery) following minimally invasive pulmonary resection (Video-Assisted Thoracic Surgery (VATS) or Robotic-Assisted). Patients were followed in hospital and 1 month after surgery for procedure-related and device-related complications and presence of air leak.

**Results:**

Forty patients had VATS and 72 patients had Robotic-Assisted procedures with the majority (80/112, 71%) undergoing anatomic resection (61 lobectomy, 13 segmentectomy, 6 bilobectomy). There were no device-related adverse events. The overall morbidity rate was 41% (46/112), with major complications occurring in 16.1% (18/112). In-hospital mortality and 30-day mortality were 1.9% (2/103). The majority of intraoperative air leaks (107/133, 81%) were sealed after sealant application, and an additional 16% (21/133) were considered reduced. Forty-nine percent of patients (55/112) were free of air leak throughout the entire postoperative study period. Median chest tube duration was 2 days (range 1 – 46 days), and median length of hospitalization was 3 days (range 1 – 20 days).

**Conclusions:**

This study demonstrated that use of a biodegradable polymer for closure of intraoperative air leaks as an adjunct to standard methods is safe and effective following minimally invasive pulmonary resection.

**Trial registration:**

ClinicalTrials.gov: NCT01867658. Registered 3 May 2013.

## Background

Lung cancer remains a common and deadly problem both in the United States and worldwide [1]. When indicated, primary surgical resection remains one of the most effective treatments for lung cancer and other isolated pulmonary conditions. Limiting postoperative morbidity in patients undergoing pulmonary resection results in decreased length of stay and reduced healthcare costs.[2] One strategy to reduce postoperative complications has been through the utilization of minimally invasive surgery (MIS) approaches, such as Video-Assisted Thoracic Surgery (VATS) and Robotic-Assisted. Multiple studies have shown that MIS pulmonary resection has benefits over a traditional thoracotomy approach, such as decreased length of stay, decreased short-term postoperative pain and fewer complications [3–6]. As a result, the utilization of VATS and Robotic-Assisted for anatomic resection has steadily increased [7]. In a recent review of the voluntary Society of Thoracic Surgery (STS) general thoracic surgery database, the rate of utilization of thoracoscopic approaches for lobectomy between 2000 and 2010 was approaching nearly 50% [8].

While increasing utilization of MIS approaches reduces overall complication rate and length of stay, one of the most common adverse events following lung resection remains prolonged air leak [9, 10]. Outside of major cardiopulmonary events, air leak management is one of the most significant causes of protracted hospital stay and cost [11]. Reflective of the impact of the problem, there have been generations of research and product development in order to achieve meaningful reduction in air leak rates. Early studies utilizing both routine and selective use of fibrin-based sealants showed no difference in duration of leak, chest tube duration, or length of stay [12–14]. The next generations of sealants were based on the use of a synthetic, water-soluble polyethylene glycol (PEG) derivative. The first approved by the FDA utilized a PEG gel applied to the lung surface and photopolymerized for postoperative pneumostasis and was shown in a multicenter, prospective randomized trial to be associated with a higher rate of patients remaining free of air leaks postoperatively [15]. The product is no longer available in the United States.

Subsequently, a polymeric biodegradable sealant that did not require light activation was developed by combining a PEG-based crosslinker, functionalized with succinate groups (PEG-(SS)_2_), with human serum albumin-USP just prior to usage (Progel™ Pleural Air Leak Sealant (PALS), Bard Davol, USA) [16]. Once mixed, Progel™ PALS polymerizes to form a clear, flexible hydrogel matrix that adheres to the lung tissue within 15 s and forms a flexible seal that can withstand 30 mmHg air pressure within 2 min of application and a maximum burst pressure of greater than 90 mmHg in less than 10 min. The material is completely reabsorbed within 1 month postoperatively. When Progel™ PALS was evaluated in a multicenter, prospective randomized trial for treatment of intraoperative air leaks following open pulmonary resection, its use was associated with a higher rate of intraoperative sealing, a lower rate of postoperative air leaks, and reduced hospital stay [17]. Based on these data, Progel™ PALS remains the only FDA-approved pneumostatic agent.

Despite data showing the efficacy of Progel™ PALS in reducing intraoperative and postoperative air leaks following open pulmonary resection, there are little data on its use during minimally invasive approaches, such as VATS or Robotic-Assisted procedures. The purpose of this multicenter, prospective study was to evaluate the safety and efficacy of Progel™ PALS for sealing air leaks incurred during MIS pulmonary resection.

## Methods

This study was conducted in compliance with the United States Code of Federal Regulations (CFR), 21 CFR Part 812, Investigational Device Exemptions, Part 56, Institutional Review Boards, Part 50, Protection of Human Subjects, and the ethical principles that have their origin in the Declaration of Helsinki. Institutional review boards at each institution approved the clinical trial prior to initiation of the study. Informed consent was obtained in writing from all patients.

Patients 18 years old and older, who were scheduled for a MIS lung resection (wedge resection, lung volume reduction surgery, segmentectomy, lobectomy, or bilobectomy), decortication, or biopsy by VATS or Robotic-Assisted approach and who gave informed consent, were evaluated for entry into the study. Patients had to have at least one visible intraoperative air leak (IOAL) at the completion of the procedure following standard closure methods, including but not limited to suturing or stapling. Exclusion criteria included: pregnancy, breast feeding, known sensitivity to human albumin, history of a previous lung resection or previous use of sealant for air leaks, renal insufficiency with a baseline serum creatinine ≥2.5 mg/dl or active dialysis, active or latent infection which was systemic or at the intended surgical site, inability to apply standard closure methods, or the presence of a significant clinical disease or condition that might complicate the surgery and make it difficult to evaluate the safety and effectiveness of the sealant.

The study was performed at 15 institutions with a mixture of academic, teaching, and community hospitals. There was a single principal investigator at each site, along with sub-investigators, and each underwent training in preparation and use of the sealant prior to the start of the study. A maximum of 20 subjects were treated at each site. Enrollment was balanced with 40 subjects undergoing VATS and 72 subjects undergoing Robotic-Assisted procedures.

The primary objective was to assess the safety of using the sealant after MIS pulmonary resection, and the primary endpoint was to measure the overall and major postoperative morbidity rates. Complications were considered as a composite rate of device- and procedure-related events and were graded according to the NCI Common Terminology Criteria for Adverse Events (Version 4.03) upon study completion. Prolonged air leak was defined as air leak present after the 5^th^ postoperative day. The secondary objective was to measure efficacy of the sealant for reducing intraoperative and postoperative air leaks. Exploratory endpoints included: the proportion of intraoperative air leaks sealed or reduced; the proportion of patients free of air leaks immediately following surgery in the recovery room; chest tube duration; and length of hospitalization.

**Fig. 1:**
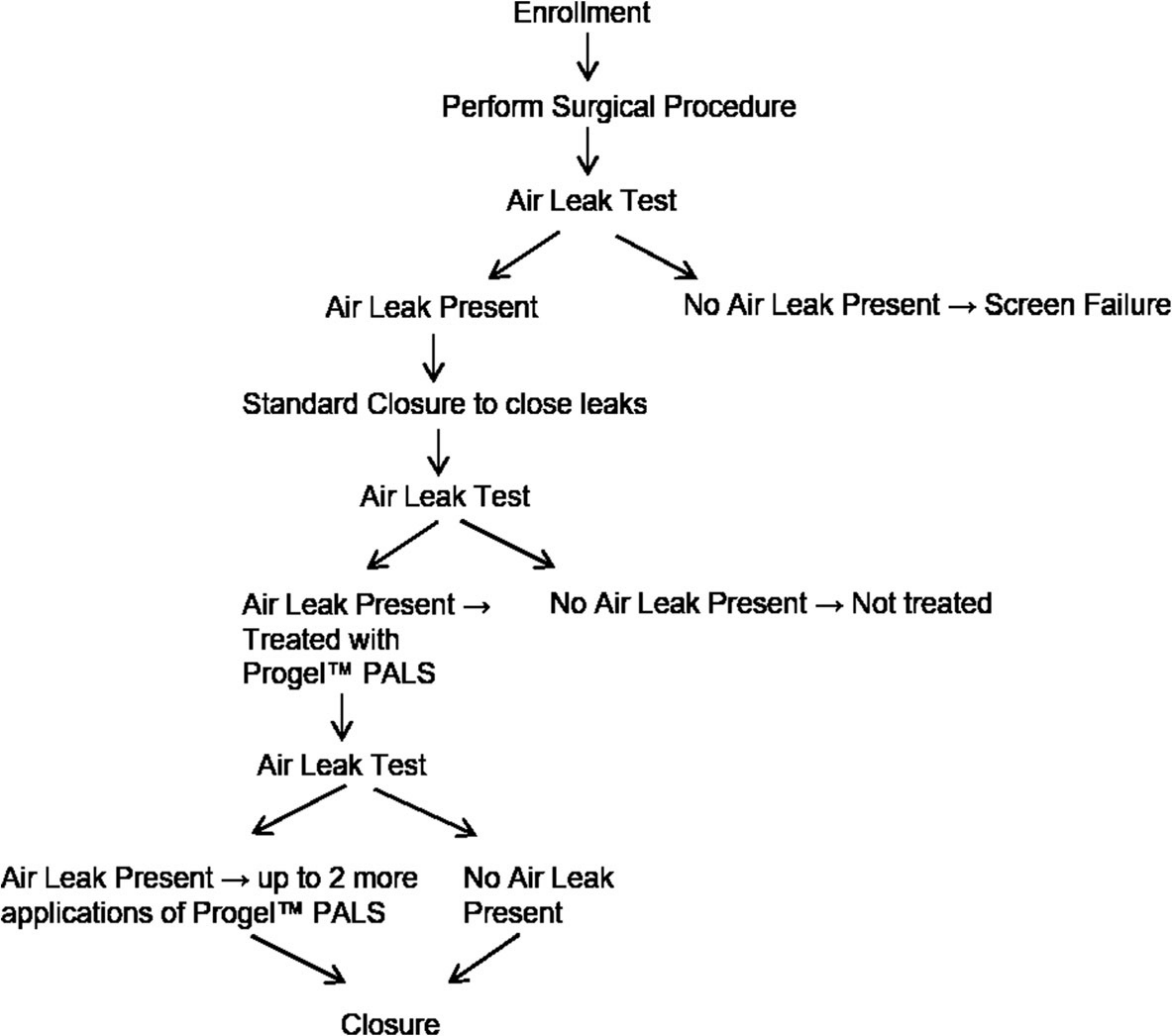
Intraoperative Protocol Schematic

After all pulmonary resections were completed, air leaks were identified by inflating the lung and submerging the areas of closure within saline solution (or water) to observe for air bubbles. If there were no air leaks detected, the patient was not treated. If air leaks were detected, standard methods of closure, such as additional suturing and stapling, were utilized, and a second leak test was performed. If there were air leaks following standard closure, the location and size of leaks were recorded, and the areas were treated with sealant. After application of the sealant to each of the identified air leaks, ventilation to the treated lung was suspended or reduced for 2 min, a second leak test was then performed, and the existence, location, and size of leaks were again recorded. If an air leak was still present, the investigator was permitted to reapply sealant up to two more times or use other closure methods (e.g., additional sutures or staples, pleural tent, pneumoperitoneum) to close the remaining air leak. Sealant was not applied prophylactically to areas of the lung that were not leaking air at the time of surgery (Fig. 1).

Kits containing the sealant were stored at 2 to 8 °C prior to usage. Each kit included two glass cartridges (one with human serum albumin-USP and the other with powdered crosslinker PEG), a syringe, and a vial of sterile water to rehydrate the powdered crosslinker just prior to usage. A double-barreled applicator was used for housing the two cartridges with a special tip to facilitate mixing of the components and to spray the mixture onto the lung. Each kit was supplied sterile and contained 4 mL of sealant.

Chest tubes were placed on 20 to 25 cm H_2_O suction for 24 h, after which they were placed to water seal if no air leak was detected. If an air leak remained after 24 h, the switch to water seal was done at the discretion of each investigator. The use of digital, regulated intrapleural pressure drains were allowed. Chest tubes were removed when the following occurred: there was no air leak following the switch to water seal; the lung had expanded sufficiently, or in the investigator’s opinion, there was no significant increase in the size of a pneumothorax that would prevent removal; and volume of drainage <5 cc/kg/24 h or <2.5 cc/kg/12 h. The duration of Post Operative Air Leak (POAL) was measured from the day of surgery until the chest tube was removed. Air leaks were assessed by qualified hospital staff, including investigators, nurses, study coordinators, physician assistants, and residents. If there were multiple readings at a designated air leak assessment time and a discrepancy was observed between readings, the investigator’s assessment was utilized. Chest roentgenograms (CXR) were obtained preoperatively, within 6 h of surgery, within 24 h prior to and after chest tube removal, and at 1-month follow-up. Some patients who had a prolonged air leak were discharged from the hospital with the chest tube connected to a Heimlich or Pneumostat™ valve. When this occurred, the patient was asked to return weekly for air leak assessment until the chest tube was removed. Chest tube duration was measured from the day of surgery to the day the last chest tube was removed. Patients were followed up at 1 month where they had a physical examination, CXR, and blood tests (blood urea nitrogen, creatinine, glomerular filtration rate) and were questioned about complications since discharge from the hospital.

A Clinical Events Committee (CEC) comprised of independent lung surgeons specializing in minimally invasive surgery, adjudicated all adverse events in this study for seriousness and for device and procedure relationship. All protocol-required CXRs were evaluated by an independent core laboratory for changes in lung expansion, presence or absence of pneumothorax, as well as size of pneumothorax.

Statistical analysis was performed using SAS software version 9.3 in accordance with the trial statistical analysis plan. All analyses were based on the modified intent-to-treat population that included those subjects who underwent successful VATS or Robotic-Assisted procedure and were treated with sealant. The key secondary endpoint of the study that measures the effectiveness of the treatment was the proportion of subjects without postoperative air leaks following lung surgery up to 1-month follow-up. The study was designed to treat approximately 105 subjects in order to obtain 100 evaluable subjects followed through the 1-month follow-up visit after allowing for 5% loss to follow-up.

## Results

**Fig. 2:**
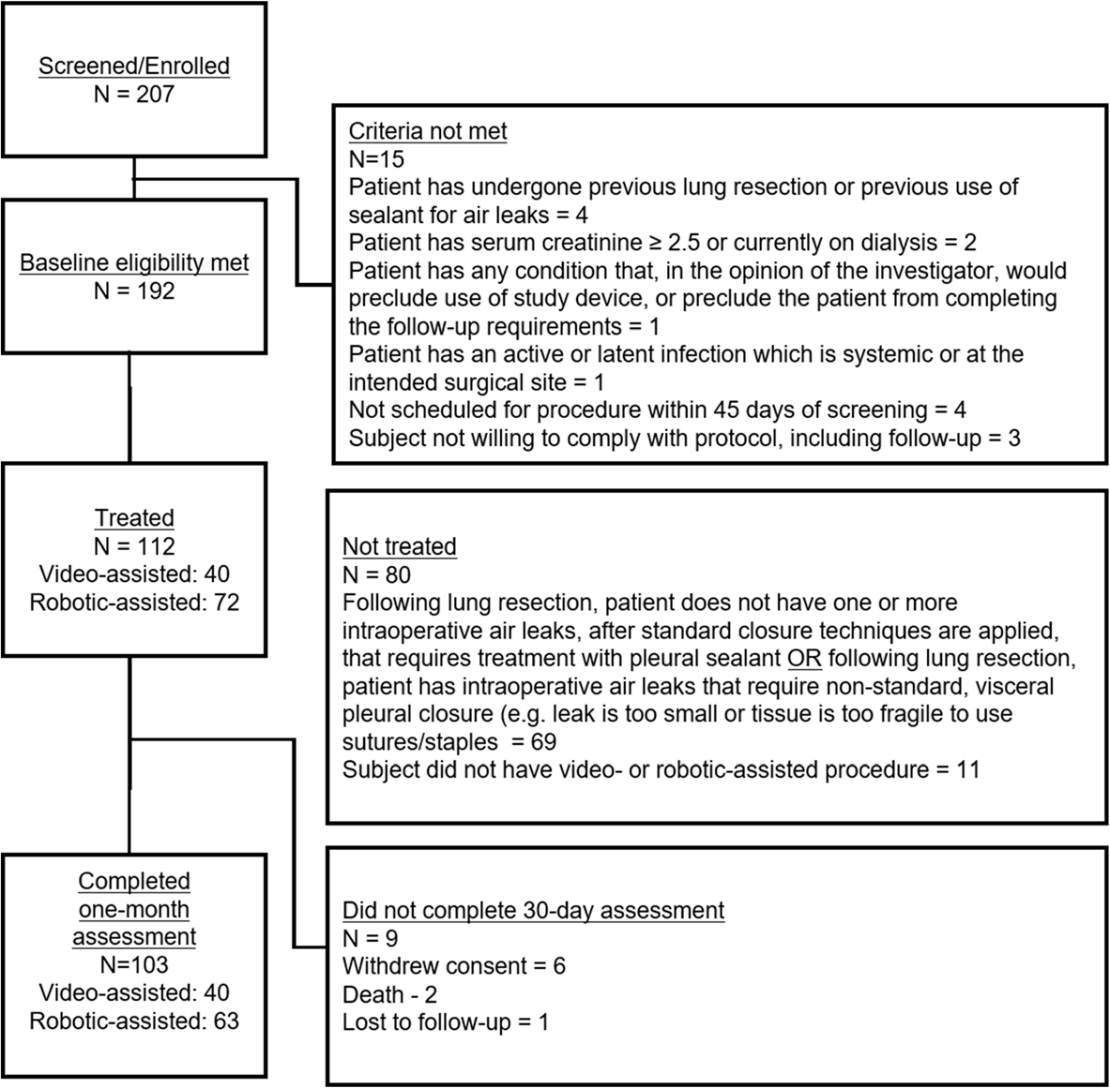
Disposition of patients and primary reasons patients were screened but not treated

There were 207 patients screened for participation, and 192 who met baseline eligibility were enrolled. There were 112 patients treated on protocol, and 80 who were not treated. The reasons for not treating these patients are listed in Fig. 2. The most common reason (*n* = 69) was either absence of intraoperative air leak following standard methods of closure or inability to utilize standard closure techniques. All 112 patients were tracked until discharge; however, 9 patients did not complete the trial through the 30-day postoperative time-point following treatment (Fig. 2).

There were 66 women and 46 men evaluated. Demographics and clinical characteristics are listed in Table 1. In general the group had good preoperative lung function, and the most common indication for surgery was a primary lung tumor. The surgical procedures performed are listed in Table 2. The most common operation performed was lobectomy (61/112), and overall anatomic lung resection was performed in 71% of patients (80/112). Eleven patients underwent more than one pulmonary procedure, but only the most extensive resection was counted. More patients underwent Robotic-Assisted procedures (72 versus 40), and a slightly higher proportion of Robotic-Assisted procedure patients underwent anatomic pulmonary resection 54/72 (75%) compared with VATS patients 26/40 (65%). There were 133 identified air leaks, among 112 subjects. In the majority of cases only one application of sealant was required to treat identified intraoperative air leaks 108/133 (81.2%).

**Table 1:** Patient Characteristics

Characteristic	All Patients (*n* = 112)	Percent (%)
Gender		
Male	46	41
Female	66	59
Median age in years (range)	69 (34–87)	
Comorbid Conditions		
Hypertension	64	
Diabetes	17	
Renal disease	10	
Previous myocardial infarction	7	
Cardiovascular disease	37	
History of stroke	4	
History of transient ischemic attack	4	
Congestive heart failure	5	
COPD	34	
Alcohol abuse (Past and Current)	11	
Diagnosis/Indications for surgery		
Primary tumor	96	86
Metastatic tumor	5	4
Lung nodule	7	6
Other^a^	4	4
Smoking History		
Never	31	27
Current	21	19
Former	60	54
Pulmonary function tests (*n* = 101)		
FEV_1_ % predicted (mean ± SD)	81.2 ± 24.3	
FVC % predicted (mean ± SD)	86.0 ± 25.5	
DLCO % predicted (mean ± SD)	70.9 ± 25.0	

*COPD* chronic obstructive pulmonary disease, *FEV*_*1*_ forced expiratory volume in 1 s, *FVC* forced vital capacity, *DLCO* diffusing capacity of carbon monoxide

^a^Other diagnosis include: COPD, chronic bronchitis, aspergilloma, bleb

**Table 2:** Perioperative Details

	*n* = 112	VATS (*n* = 40)	Robotic-Assisted (*n* = 72)
Operative procedures^a^			
Wedge resection	29	14	15
Segmentectomy	13	4	9
Lobectomy	61	21	40
Bilobectomy	6	1	5
Decortication	2	0	2
Biopsy	1	0	1
Median OR time, minutes (range)	214.5 (79 – 433)		

*VATS* video-assisted thoracic surgery, *OR* operating room

^a^Eleven subjects had more than one procedure; only the most extensive procedure was counted

### Morbidity

There were no observed device-related adverse events. The overall complication rate for the entire treated group was 41% (46/112). Major complications, as defined as NCI CTCAE (v4.03) [18] grade 3 or higher adverse events, occurred in 18 patients (16.1%) (Table 3). Prolonged air leak was the most common single major adverse event, occurring in 10 patients (8.9%). Of the 10 patients with prolonged air leak, six were considered major because they required additional intervention, such as insertion of an additional chest tube or chemical pleurodesis. Two such patients underwent reoperation — one had bleb resection, and one had creation of a pleural tent. An additional 5 patients underwent reoperation for indications including postoperative hemorrhage, pulmonary torsion, and repair of bronchopleural fistula, for a total reoperation rate of 6.3% (7/112).

**Table 3:** Major Complications^a^

Prolonged air leak	6
Postoperative hemorrhage	3
Pleural effusion	2
Acute respiratory distress syndrome	2
Pneumothorax	1
Pulmonary torsion	1
Bronchopleural fistula	1
Pericardial effusion	1
Subcutaneous emphysema	1
Aspiration pneumonitis	1
Cardiac arrest	1
Pneumonia	1
Positive resection margin	1
Multi-organ failure	1
Total	23

Terminology Criteria for Adverse Events (v4.03)

^a^Major complications defined as Grade 3 or higher as per National Cancer Institute Common

In-hospital mortality and 30-day mortality were each 1.9% (2/103). One patient underwent Robotic-Assisted lobectomy and developed right upper lobe torsion necessitating reoperation. Postoperatively the patient suffered sudden cardiac arrest and expired on postoperative day 13. The second patient underwent Robotic-Assisted lobectomy and suffered aspiration pneumonitis on postoperative day 2. This progressed to acute respiratory distress and multi-system organ failure. The patient had withdrawal of care and died on postoperative day 11.

There were a total of 133 intraoperative air leaks identified in 112 patients (Table 4). The most common site of leak was from the area of the staple line (98/133, 74%). The median number of applications of sealant was 1 (range 1–3), and 80% (107/133) of leaks were sealed at the time of operation, while another 21 (16%) were reduced. Postoperatively, 61% of patients had no leak in the post-anesthesia care unit, and 49% of patients demonstrated no air leak up through the 1 month postoperative visit (Table 5). The median chest tube duration was 2 days (range 1 – 46), and the median length of stay for the entire cohort was 3 days (range 1 – 20). Eight patients were discharged with a chest tube in place and had removal in the outpatient setting.

**Table 4:** Intraoperative Air Leaks (IOAL)

Variable	Number of Air Leaks (*n* = 133)	Percent (%)
Source of air leak		
Suture line	9	6.7
Staple line	98	73.8
Lung surface	12	9.0
Torn lung	5	3.8
Fissure	9	6.7
Final assessment of IOAL		
Not sealed	5	3.8
Reduced	21	15.8
Sealed	107	80.4

**Table 5:** Comparative Efficacy of Progel™ PALS in MIS and Open Studies

	Current Study	Progel™ PALS Open Study [17]	
Endpoint	MIS Treatment Group	Treatment Group	Control
Air leaks sealed in the OR	80%	77%	16%
Air leaks sealed immediately following surgery	60.7%	54%	33%
Air leaks sealed through 1 month post-operative	49.1%	35%	14%
Median duration of post-op air leakage	1.0 day	2 days	2 days
Median chest tube duration	2.0 days	5 days	5 days
Median length of stay	3.0 days	6 days	7 days

*MIS* minimally invasive surgery, *PALS* pleural air leak sealant, *OR* operating room

## Discussion

This is the first study, prospective or otherwise, evaluating the feasibility of utilizing lung sealant following minimally invasive lung resection. It demonstrated that the use of Progel™ PALS was safe, with no device-related morbidity and with overall major procedural morbidity rates that were consistent with similar large series of VATS and Robotic-Assisted lung resections [3, 4, 6, 7]. Boffa and co-authors recently reported results of a retrospective study of VATS versus open anatomic lung resections from 11,531 patients in the voluntary Society of Thoracic Surgeons database [19]. The overall complication rate for VATS was 30%, with an operative mortality rate of 1.3%. By comparison, in our study the overall morbidity was 41% with an operative mortality rate of 1.9%. Taking into account the prospective nature of the current study, the results are acceptable. In addition, the observed chest tube duration and length of stay were substantially shorter than the comparable data from the previous randomized trial (Table 5).

To date, this is the only experience in a cohort undergoing exclusively minimally invasive thoracic surgery. The previous prospective, randomized trial that demonstrated efficacy of this solitary FDA-approved product to seal intraoperative air leaks and reduce length of stay was almost exclusively in patients undergoing thoracotomy [17]. Two other prospective, randomized studies of an alternative polyethylene glycol matrix (currently not approved for use in the United States) were conducted as adjuncts to predominantly open approaches [20, 21]. In the more recent report, while the authors stated that some proportion of procedures were performed by VATS, they did not report the distribution of surgical approach [21].

The secondary endpoints observed regarding the efficacy of the sealant were also promising and consistent with the prior efficacy study (Tables 4 and 5). Intraoperative air leaks were sealed (21/133, 15.8%) or reduced (107/133, 80.4%) in 96.2% of cases, and 49% of the patients had no evidence of air leak through 1-month of postoperative follow-up. This compares favorably with the results reported by Allen and colleagues, who observed that 35% of patients experienced freedom from air leak throughout the entire 1-month follow-up, versus only 14% of the control group. Similarly, Klijian performed a retrospective review of a single institution experience comparing patients undergoing lung resection who received intraoperative pleural sealant with those who did not [22]. In individuals with intraoperative air leak, the use of sealant was associated with a significant decrease in the incidence of postoperative air leak (11% in the sealant group versus 58.8% in the control group, *p* <0.0001) as well as shorter chest tube duration and hospital stay.

The strengths of the current study include the prospective multicenter design, rigorous prospective documentation of potential product- and procedural-related complications, and the standardization of intraoperative air leak assessment and subsequent treatment. In particular, the requirement that patients must have a demonstrable intraoperative air leak to be treated, while not necessary to determine the safety of the product, eliminated unnecessary treatment and allowed for evaluation of secondary efficacy outcomes.

There are some limitations of this report. The treatment population was somewhat heterogeneous, with a mixture of patients undergoing non-anatomic and anatomic resection, as well as different minimally invasive approaches (VATS and Robotic-Assisted). Moreover, without a control group undergoing only standard methods to prevent air leak, conclusions about the efficacy of the sealant by comparison to historical data is limited. A follow-up study utilizing a prospective, randomized case-control methodology to more rigorously evaluate efficacy would be ideal. Included in the study should be a planned cost-effectiveness analysis as well, to determine whether any potential clinical benefit also has an acceptable cost profile.

## Conclusions

The use of PEG cross-linked with human serum albumin as a pneumostatic agent for patients with intraoperative air leak following minimally invasive pulmonary resection is safe and efficacious. In addition, its use does not appear to be associated with increased procedural morbidity or mortality. Based upon the results of the study, the product’s use was observed to reduce intraoperative air leak and should be considered for use on pleural air leaks identified during lung resection in open or minimally invasive procedures.

## Abbreviations

CXR: Chest roentgenogram
MIS: Minimally invasive surgery
VATS: Video-assisted thoracic surgery
STS: Society of thoracic surgery
PEG: Polyethylene glycol
FDA: Federal drug administration
IOAL: Intraoperative air leak
POAL: Postoperative air leak
AEs: Adverse events
PG: Performance goal
CFR: Code of federal regulations
NCI CTCAE: National cancer institute common terminology for clinical adverse events
